# Comprehensive Definition of the SigH Regulon of *Mycobacterium tuberculosis* Reveals Transcriptional Control of Diverse Stress Responses

**DOI:** 10.1371/journal.pone.0152145

**Published:** 2016-03-22

**Authors:** Jared D. Sharp, Atul K. Singh, Sang Tae Park, Anna Lyubetskaya, Matthew W. Peterson, Antonio L. C. Gomes, Lakshmi-Prasad Potluri, Sahadevan Raman, James E. Galagan, Robert N. Husson

**Affiliations:** 1 Division of Infectious Diseases, Boston Children’s Hospital and Department of Pediatrics, Harvard Medical School, Boston, Massachusetts 02115, United States of America; 2 National Emerging Infectious Diseases Laboratories, Boston University, Boston, Massachusetts 02118, United States of America; 3 Bioinformatics Program, Boston University, Boston, Massachusetts 02215, United States of America; 4 Department of Biomedical Engineering, Boston University, Boston, Massachusetts 02215, United States of America; 5 Department of Microbiology, Boston University, Boston, Massachusetts 02215, United States of America; Indian Institute of Science, INDIA

## Abstract

Expression of SigH, one of 12 *Mycobacterium tuberculosis* alternative sigma factors, is induced by heat, oxidative and nitric oxide stresses. SigH activation has been shown to increase expression of several genes, including genes involved in maintaining redox equilibrium and in protein degradation. However, few of these are known to be directly regulated by SigH. The goal of this project is to comprehensively define the *Mycobacterium tuberculosis* genes and operons that are directly controlled by SigH in order to gain insight into the role of SigH in regulating *M*. *tuberculosis* physiology. We used ChIP-Seq to identify *in vivo* SigH binding sites throughout the *M*. *tuberculosis* genome, followed by quantification of SigH-dependent expression of genes linked to these sites and identification of SigH-regulated promoters. We identified 69 SigH binding sites, which are located both in intergenic regions and within annotated coding sequences in the annotated *M*. *tuberculosis* genome. 41 binding sites were linked to genes that showed greater expression following heat stress in a SigH-dependent manner. We identified several genes not previously known to be regulated by SigH, including genes involved in DNA repair, cysteine biosynthesis, translation, and genes of unknown function. Experimental and computational analysis of SigH-regulated promoter sequences within these binding sites identified strong consensus -35 and -10 promoter sequences, but with tolerance for non-consensus bases at specific positions. This comprehensive identification and validation of SigH-regulated genes demonstrates an extended SigH regulon that controls an unexpectedly broad range of stress response functions.

## Introduction

*Mycobacterium tuberculosis* is a slow-growing but deadly pathogen that is able to cause a chronic infection through its ability to adapt to the multiple environments that it encounters in the human host during infection. During the course of *M*. *tuberculosis* infection, ranging from primary infection with active bacterial replication, to latent infection with restricted replication within granulomas, to cavitary tuberculosis (TB) where many bacteria are extracellular, *M*. *tuberculosis* is subject to an extensive and varying array of host-generated stresses. Among these are hypoxia, and several forms of oxidative and nitrosative stresses that can damage a broad range of bacterial macromolecules that are essential for viability [1, 2]. For *M*. *tuberculosis* to persist in the face of these stresses requires extensive defense and repair mechanisms, many of which result from changes in gene expression in response to environmental cues.

The *M*. *tuberculosis* genome encodes 13 sigma factors, the subunit of RNA polymerase that binds specific promoter sequences to initiate transcription [3]. In addition to SigA, the primary sigma factor that controls the expression of a large proportion of genes under most conditions, the *M*. *tuberculosis* genome encodes 12 alternative sigma factors that respond to specific signals to regulate the transcription of genes and operons that are physically separated but functionally linked [4, 5]. Ten of these sigma factors belong to the Type 4 or extracytoplasmic function (ECF) family, [6, 7]. The activity of the ECF sigma factors is often regulated by reversible binding of the sigma factor by a cognate anti-sigma factor, which functions as a sensor of signals from the environment [8, 9].

SigH is an ECF sigma factor of *M*. *tuberculosis* that is activated by heat stress, oxidative stress and nitric oxide stress. SigH activity is regulated post-translationally by its anti-sigma factor, RshA, which senses these stresses and releases SigH to bind to core RNA polymerase and activate transcription of its regulon [10–13]. This sigma factor is required for full virulence of *M*. *tuberculosis* in mouse and non-human primate (NHP) models of infection [14, 15]. In primate macrophages SigH is required for long-term infection and has been shown to affect the expression of host genes in response to *M*. *tuberculosis* infection, with effects on host cell apoptosis [16]. Based on potent immunity in a non-human primate model, a *sigH* deletion strain of M. tuberculosis has been suggested to be a candidate for development of vaccines to protect against M. tuberculosis disease [17]. Analysis of a *sigH* mutant *M*. *avium* subsp. paratuberculosis infection of bovine macrophages and a murine model of paratuberculosis suggested that *sigH* could play an important role in persistence and virulence of this mycobacterial species in infected animals [18].

Despite its importance for virulence and host responses to infection, only a limited number of genes have been experimentally shown to be directly regulated by SigH [11, 19]. These genes include SigH itself and additional transcriptional regulators, heat shock genes required for repair and degradation of damaged proteins, and components of the thioredoxin/thioredoxin reductase system required to maintain redox homeostasis. In addition to these genes that are known to be directly regulated by SigH, increased stress-induced expression of several additional genes in wild type *M*. *tuberculosis* compared to *sigH* deletion strains has been observed [12, 14]. These data suggest that the direct SigH regulon may be substantially larger than is currently known. Because SigH regulates expression of other transcription regulators, however, including SigE and SigB, two sigma factors that also regulate stress responses, differential gene expression may result from indirect effects via these SigH-controlled regulators or by SigH-mediated changes in cell physiology that affect the activity other transcription factors.

We therefore undertook to comprehensively define the direct SigH regulon of *M*. *tuberculosis*, using chromatin immunoprecipitation with massively parallel sequencing (ChIP-Seq) to identify DNA regions bound by this sigma factor *in vivo* in the *M*. *tuberculosis* cell. We then compared the stress-induced expression in *M*. *tuberculosis* wild type and Δ*sigH* strains of candidate SigH-controlled genes identified by ChIP-Seq. For genes where we observed differential gene expression between these strains, we performed 5’-RACE using RNA from wild type and Δ*sigH* strains, to identify SigH-dependent transcription start points (TSPs). From these data we derived a highly refined SigH-regulated promoter motif consensus.

Defining genes directly regulated by SigH as those i) that have a sequence bound by SigH, ii) that show differential gene expression in wild type versus Δ*sigH* strain and iii) that have a promoter sequence consistent with the SigH consensus, our data identify a minimum of 25 genes that comprise the direct SigH regulon. The experimentally defined or predicted functions of these genes indicate that in addition to regulation of redox homeostasis and protein turnover, SigH plays a much broader role in recovery from stresses than was previously known, including regulation of genes required for repair of DNA damage, recovery of ribosome function and translation, sulfur transport, and synthesis and salvage of sulfur-containing amino acids.

## Materials and Methods

### Chromatin Immunoprecipitation and Sequencing (ChIP-Seq)

ChIP-Seq was performed as previously described with minor modifications [20]. The *sigH*-FLAG fusion was expressed using a tetracycline repressor (TetR)-regulated promoter [21]. Prior to performing the ChIP-Seq experiments, the expression of SigH-FLAG was analyzed to determine the optimal concentration of the anhydrotetracycline (aTc) inducer and the optimal time point following induction. Based on these experiments, *M*. *tuberculosis* was grown at 37°C in Middlebrook 7H9 broth (Becton Dickinson) supplemented with ADC [albumin (50 g l-1), dextrose (20 g l-1), NaCl (8.1 g l-1)], hydrolyzed casein (1g l-1), 0.2% glycerol, and 0.05% Tween 80. At OD_600_ = 0.5, aTc was added to the culture at a final concentration of 200 ng/ml and cells were grown for 24h, followed by cross-linking with formaldehyde, quenching, pelleting, cell lysis, DNA shearing, immunoprecipitation with anti-FLAG antibody and further processing as described [20].

Genomic DNA enriched for SigH binding was then sequenced on the Illumina platform using a GAIIx instrument (Boston University sequencing core facility). Significantly enriched peaks in the sequence data from the SigH-FLAG-expressing strain relative to the vector control strain were identified as previously described [22]. Briefly, coverage along the genome was calculated using Bowtie2 [23] and SamTools [24]. The total coverage at each position of the genome was calculated and significantly enriched regions (P<0.01) were called using log-normal distributions as described previously [20]. Continuous regions of enriched coverage that were 150 base pairs or more in length were selected for further analysis. In addition, a shift of at least 60 nucleotides was required between the peak in forward and reverse read coverage, as assessed by a cross-correlation. Region coverage was normalized using mean coverage of an experiment, correcting for differences in the number of reads among experiments. Exact binding sites were determined as described by [25], and motifs present in the identified sites were determined using MEME [26, 27].

The *sigH*-FLAG fusion was expressed in either wild type *M*. *tuberculosis* H37Rv or a Δ*sigH* strain derived from H37Rv [11]. Data from an independent experiment in each strain expressing *sigH*-FLAG were compared to results from the Δ*sigH* strain containing the vector only and significantly enriched sites were identified as described above. In addition to the SigH binding sites identified using the statistical approaches described above, additional candidate SigH binding sites were identified by visual inspection of tdf files displayed using the Integrative Genomics Viewer [28]. Putative SigH target sequences were associated with binding sites based on relative location. Intergenic binding sites were provisionally assigned possible regulatory function for the immediate up- and downstream genes oriented away from the binding sites. Binding sites within a gene were provisionally associated with the gene itself or an immediately adjacent gene if oriented away from the binding site.

### RNA Isolation and quantitative reverse transcription polymerase chain reaction (qRT-PCR)

To evaluate whether binding sites identified by ChIP-Seq were linked to genes that showed SigH-dependent transcription, we performed quantitative reverse transcription PCR (qRT-PCR). H37Rv and Δ*sigH* strains were grown to mid-log phase (OD_600_ = 0.4–0.5) at which point the cultures were subjected to heat stress at 52°C for 15 min. To assess transcription following oxidative stress, both strains were grown to mid-log phase and exposed to 50μM plumbagin for 20 minutes. Cells were then harvested by centrifugation, re-suspended in TRI Reagent (Molecular Resource Center) and mechanically disrupted (Magna Lyser, Roche). RNA was extracted according to the manufacturer’s protocol and purified using an RNA cleanup kit (Qiagen), followed by two rounds of DNAse treatment. The quantity and quality of RNA was determined by measuring absorbance at 260 and 280 nM using a Nanodrop instrument (Thermo Fisher). Reverse transcription was performed using the qScript cDNA synthesis kit (Quanta Bioscience).

Genes were selected for transcript quantification if they were adjacent to and oriented away from the SigH binding sites identified in the ChIP-Seq experiments. If a binding site was within the 5’ half of the annotated coding sequence of a gene the expression of the 3’ region of that gene was quantified. For some intragenic binding sites, we also determined whether a SigH-regulated antisense transcript was present. A map of all evaluated genes is shown in supplemental **S1 Fig**. qRT-PCR of cDNA was performed by using the PerfeCTa SYBR Green Supermix (Quanta Biosciences) on an Applied Biosystems 7300 real time PCR system using the following protocol: denaturation at 95°C for 3 min., followed by 40 cycles of denaturation at 95°C for 15 s and annealing/elongation at 60°C for 1 min. Data were analyzed using the ΔΔ^CT^ method using the housekeeping gene *sigA* as the control [29]. The initial reactions were performed in technical duplicates and all genes with an expression ratio (wild type:Δ*sigH*) >2 were analyzed in two biological replicates. The primers used for qRT-PCR are shown in **S1 Table**.

### Identification of transcription start points and determination of a consensus SigH-regulated promoter sequence

Genes that were determined to have >2-fold higher expression in wild type relative to the Δ*sigH* strain following heat stress were selected for experimental determination of transcription start points (TSPs). RNA Ligase Mediated-Rapid Amplification of cDNA Ends (5’-RLM-RACE) was performed on RNA extracted from H37Rv and the Δ*sigH* strain following 52°C heat stress as described above. First, Terminator 5’-phosphate dependent exonuclease (Epicentre) was used to digest the total RNA sample containing mRNAs, rRNAs and tRNAs to produce an mRNA-enriched preparation. RNA 5’-polyphosphatase was then used to convert 5´-triphosphorylated RNA (mRNA) into 5´-monophosphorylated RNA, which was then used for 5’ RACE adapter ligation. The resulting 5’ adapter-ligated mRNA was then used for cDNA synthesis (qScript, Quanta Bioscience) and this cDNA was then used as the template for amplification of specific genes. Two rounds of PCR were performed, using outer and then inner 3’ primers specific for each gene, with an adapter-specific 5’ primer in each reaction. Amplicons from the inner PCR were run on an agarose gel. PCR products present in reactions using RNA from wild type, but absent in reactions using RNA from the Δ*sigH* strain were gel-purified and cloned into pGEM-T (Promega). In cases where PCR products from H37Rv and Δ*sigH* only had amplicons of similar size, the amplicon from the wild type reaction was selected. The cloned PCR products were sequenced to identify the TSP as the locus-specific base immediately 3’ to the adapter sequence. The adapter sequence and the primers used for 5’-RACE experiments are shown in **S2 Table**.

The 50 bases 5’ of each TSP identified experimentally by 5’-RLM RACE, together with SigH-regulated promoters that had been previously identified experimentally, were analyzed using the MEME algorithm [27], to determine conserved sequences among these promoter regions.

## Results

### Genome-wide identification of SigH binding sites

**Fig 1:**
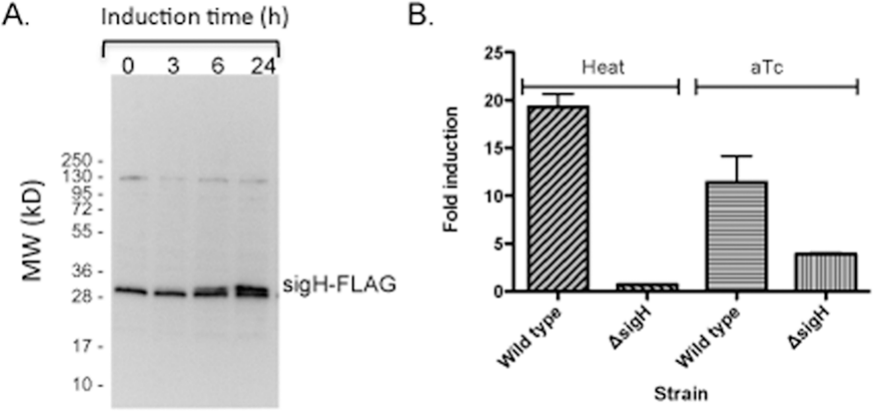
Expression of *sigH* and *sigH*-FLAG in *M*. *tuberculosis*. **A.** *sigH*-FLAG expression was induced in *M*. *tuberculosis* H37Rv (wild type) containing a vector expressing *sigH*-FLAG under control of a TetR-regulated promoter. Samples were obtained at serial time points following addition of aTc to a final concentration of 200 ng/ml, protein was extracted and Western blotting was performed with an anti-FLAG antibody (Sigma-Aldrich). **B**. *M*. *tuberculosis* H37Rv (wild type) or the Δ*sigH* strain were exposed to 52°C for 15 minutes. The same strains containing a vector expressing *sigH*-FLAG under control of a TetR-regulated promoter were induced by addition aTc to a final concentration of of 200 ng/ml (aTc). RNA was extracted after 24h and qRT-PCR was performed and analyzed as described in the Materials and Methods. The higher level of induced *sigH* expression in wild type compared to the Δ*sigH* strain likely results from increased expression in wild type of the native copy of *sigH* from its SigH-regulated promoter following induction of the TetR-regulated copy of *sigH*.

ChIP-Seq was performed to identify the DNA sequences throughout the *M*. *tuberculosis* genome that are bound by SigH. These experiments were performed using SigH fused to the FLAG epitope tag, expressed from an inducible TetR-regulated promoter [21]. A replicating plasmid expressing *sigH*-FLAG was introduced into both H37Rv (wild type) *M*. *tuberculosis* and a Δ*sigH* strain derived from H37Rv [11]. SigH-FLAG was found to be optimally expressed at 24 hours of induction using 200 ng/ml of anhydrotetracycline (aTc), as determined by Western blotting (**Fig 1A**). As measured by qRT-PCR, the level of *sigH*-FLAG expression in H37Rv following aTc-induction is approximately 60% of the expression of the level of *sigH* from the chromosomal copy of this gene following stimulation with heat stress, suggesting that the level of induced expression would not likely result in binding of chromosomal DNA at sites that are not bound by native SigH in response to stress (**Fig 1B**). To perform the ChIP-Seq experiments, wild type *M*. *tuberculosis* H37Rv or a Δ sigH strain was grown to mid-log phase at which point expression of *sigH*-FLAG was induced by adding aTc and incubating at 37°C for 24h. DNA sites that were bound by SigH in these strains, but not in a strain in which the vector only was present, were identified using an optimized ChIP-Seq protocol as described in the materials and methods [20].

**Fig 2:**
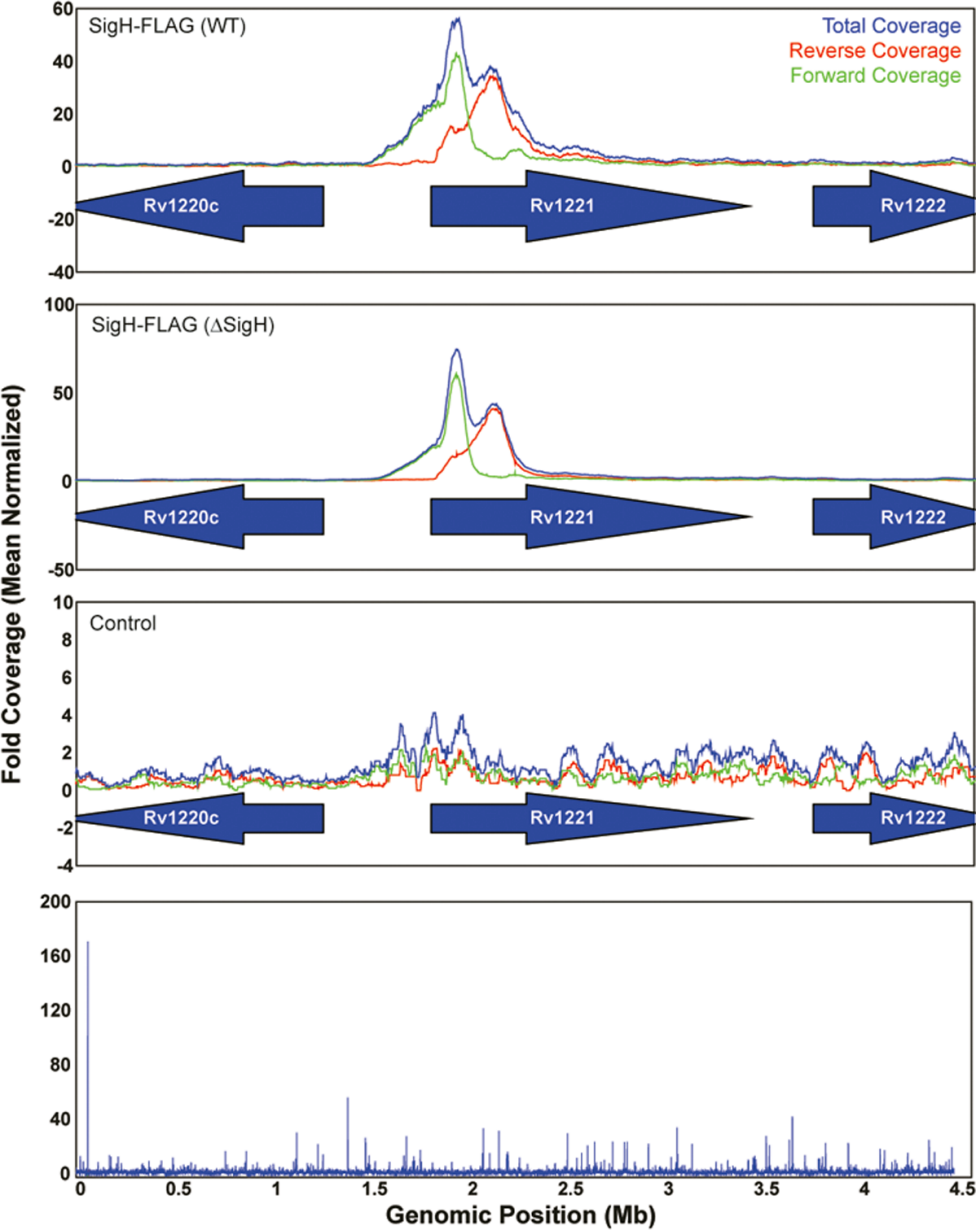
ChIP-Seq results for *M*. *tuberculosis* SigH. **A**. Sequencing read coverage for a region with a known SigH binding site 5’ of *sigE* (Rv1221) in two independent experiments. The total coverage is shown in blue, and the forward and reverse coverages are shown in red and green, respectively. The binding displays the expected shift in position between the forward and reverse reads. **B.** Genome-wide fold read coverage.

62 sites showed significant SigH binding and 7 additional candidate binding sites were identified by visual inspection of the data (**S1 Fig** and **S3 Table**). The ChIP-Seq binding data, represented graphically, are shown in **Fig 2**. The upper panel shows a representative binding peak for the region 5’ of *sigE*, which has a known SigH binding site, with the characteristic shift in the position of the sequences bound in the forward versus the reverse DNA strands. The distribution and read coverage for binding sites throughout the genome are shown in the lower panel. The ChIP-Seq experiments identified binding sites 5’ of all but one of the 7 genes previously confirmed to be directly regulated by SigH in our previous work. The exception was *clpB*, which is co-regulated by a heat responsive repressor whose binding site overlaps the SigH-regulated promoter of this gene [11, 30]. In addition, binding sites were identified that are linked to several genes identified in earlier microarray experiments that showed decreased expression in Δ*sigH* strains relative to wild type [11, 12, 14]. We also identified several binding sites linked to genes not previously shown to be SigH-regulated, suggesting a substantially expanded direct SigH regulon. Notable among these genes are *udgB*, which encodes a DNA repair enzyme, *rpmE*, which encodes a zinc-binding ribosomal protein, *msrB*, which encodes an oxidized methionine repair enzyme, molybdopterin sulfuryltransferases, a set of genes encoding a cysteine biosynthesis pathway, and several genes of unknown function.

**Fig 3:**
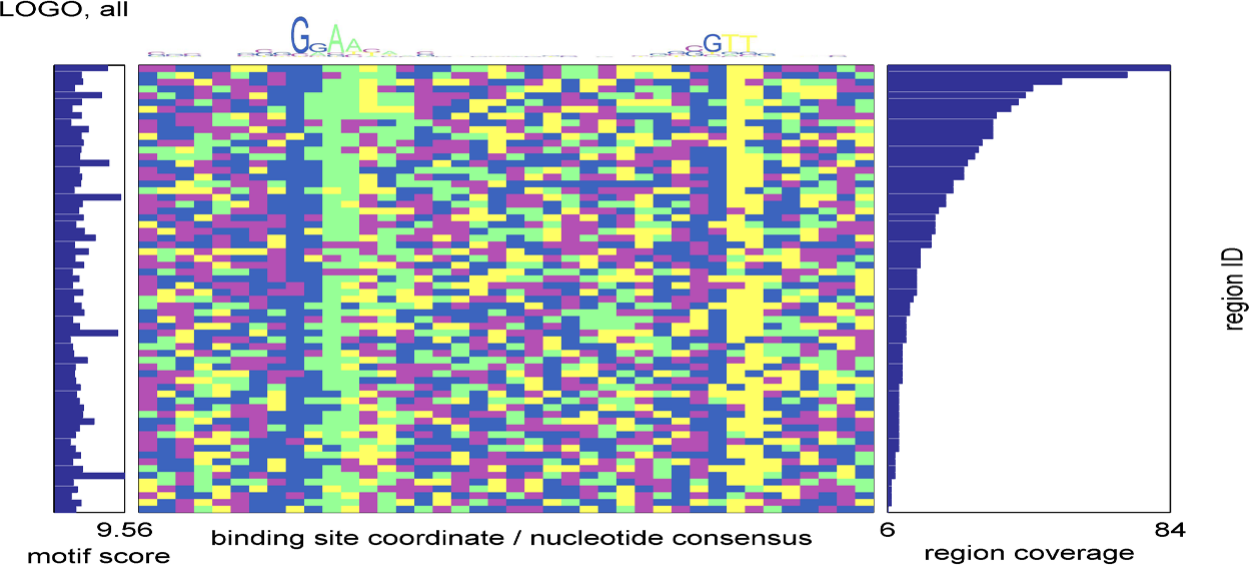
Analysis of of SigH binding sites identified by ChIP-Seq. MEME (http://meme-suite.org/) was used to predict binding motifs de novo within SigH ChIP-Seq regions. In the heatmap shown in the center panel, columns are motif positions that correspond to the LOGO at the top, and rows are sequences from the SigH ChIP-Seq binding sites. The colors of the blocks in the heatmap correspond to the colors of the bases shown in the LOGO. Sequences containing the motif are sorted according to the coverage of the corresponding ChIP-Seq region as shown in the bar-plot to the right of the heatmap. The corresponding motif score (negative log10 p-value) for each sequence is shown in the bar-plot to the left. The core GGAA of the -35 element and the cGTT of the -10 element are prominent even in this analysis of all binding sites.

The sequences corresponding to each region of DNA bound by SigH were compiled and searched for the presence of shared sequence features. As shown in **Fig 3**, this analysis identified two conserved sequences, GGAA and GTT, separated by 19 base pairs. These sequences correspond to the most highly conserved bases of the -35 and -10 elements of the consensus promoter sequence recognized by SigH that was derived from 7 genes previously shown to be directly SigH-regulated [11].

### Quantification of SigH-dependent transcription from genes adjacent to SigH binding sites

Sigma factor binding of DNA is expected to occur predominantly at promoters as part of RNA polymerase holoenzyme, resulting in transcription initiation. Previous ChIP-Seq studies in *M*. *tuberculosis* and other bacteria, however, have consistently found that not all transcription factor binding sites identified by ChIP are associated with regulation of transcription of adjacent genes under the conditions tested [31–33]. To determine which of the SigH binding sites that we identified are linked to SigH-dependent regulation of transcription, we isolated RNA from heat-stressed wild type *M*. *tuberculosis* and from the Δ*sigH* strain, and performed qRT-PCR to measure the expression of genes adjacent to SigH binding sites. For intergenic sites, we analyzed transcripts for annotated genes that were 3’ of the binding site and oriented such that the binding site could function as a promoter. For sites that occurred within annotated open reading frames we quantified transcripts within those genes and in the adjacent gene 3’ of the binding sites. For a subset of intragenic binding sites we also looked for the presence of anti-sense transcripts. The location of SigH binding sites identified by Chip-Seq and the genes analyzed by qRT-PCR are shown in **S1 Fig**.

Using this approach and averaging the expression from three biological replicates, we found that 41 of 69 unique binding sites (59%) were linked to transcripts that had more than 2-fold greater expression in wild type compared to the Δ*sigH* strain (**Table 1**). This is a higher proportion of transcription factor binding sites associated with regulated transcription compared to many other ChIP studies, e.g. 25 of 67 (37%) *M*. *tuberculosis* SigF binding sites were linked to SigF-dependent transcription in a ChIP-chip analysis, and a ChIP-Seq study of >100 *M*. *tuberculosis* transcription factors found transcription factor overexpression resulted in changes in expression of genes linked to <10% of transcription factor binding sites [31, 32]. That study identified 36 SigH binding sites, of which 10 (28%) were linked to genes that were significantly upregulated following *sigH* overexpression. We identified 26 of these binding sites in our experiments. It is not clear why we identified substantially more binding sites in our study, however, our optimization of SigH-FLAG expression together with our inclusion of a small number of manually identified sites likely contributed to the greater number of binding sites identified in this work. The several binding sites that are uniquely present in our ChIP-Seq results that are linked to SigH-regulated expression in our data and in previous studies supports the validity of our binding data (Table 1).

**Table 1:** Genes linked to SigH binding sites that show SigH-dependent expression following heat stress.

Rv No.^a^	Gene	H37Rv/Δ*sigH*^b^	Gene product
Rv0016c	*pbpA*	43.4±33.0	Probable penicillin-binding protein PbpA
Rv0100		29.7±24.4	Conserved hypothetical protein
Rv0101	*nrp*	2.5±0.2	Probable peptide synthetase Nrp
Rv0140		71.2±42.3	Conserved protein
Rv0141c		33.4±24.1	Unknown protein
Rv0303		2.7±0.8	Probable dehydrogenase/reductase
**Rv0350**	*dnaK*	21.6±9.2	Probable chaperone protein DnaK
Rv0488		34.8±11.0	Probable conserved integral membrane protein
Rv0654		4.1±0.6	Probable dioxygenase
Rv0759c		9.9±6.6	Conserved hypothetical protein
Rv0991c		40.3±12.2	Conserved serine rich protein
Rv1038c	*esxj*	4.1±2.5	ESAT-6 like protein EsxJ
Rv1039c	*PPE15*	8.3±7.9	PPE family protein PPE15
**Rv1221**	*sigE*	169.0±52.2	Alternative RNA polymerase sigma factor SigE
Rv1259	*udgB*	420.3±184.3	Probable uracil DNA glycosylase
Rv1298	*rpmE*	9.6±0.3	50S ribosomal protein L31
Rv1334	*Mec*	116.5±26.4	Possible hydrolase
**Rv1471**	*trxB1*	76.7±32.6	Probable thioredoxin
**Rv1528c**	*papA4*	5.3±1.9	Probable conserved polyketide synthase associated protein
Rv1801	*PPE29*	17.3±4.7	PPE family protein PPE29
**Rv1875**		101.0±12.9	Conserved protein
Rv2204c		36.3±7.3	Conserved protein
Rv2266	*cyp124*	2.9±0.3	Probable cytochrome P450 124 Cyp124
Rv2332	*mez*	2.1±0.5	Probable [NAD] dependent malate oxidoreductase
Rv2373c	*dnaj2*	5.7±2.5	Probable chaperone protein
Rv2386c	*mbtI*	8.1±4.2	Isochorismate synthase MbtI
Rv2387		2.3±0.3	Conserved protein
Rv2400c	*subI*	74.7±16.1	Probable sulfate-binding lipoprotein
Rv2454c		2.5±1.6	Probable oxidoreductase
**RV2466c**		516.1±103.6	Conserved protein
Rv2585c		2.4±1.1	Unknown protein
Rv2674	*msrB*	4.9±1.6	Probable peptide methionine sulfoxide reductase
Rv2706c		6.7±3.4	Hypothetical protein
**Rv2707**		11.6±4.8	Probable conserved alanine and leucine rich protein
**Rv2710**	*sigB*	15.6±8.5	RNA polymerase sigma factor SigB
Rv2906c	*trmD*	12.6±4.3	Probable tRNA (guanine-N1)-methyltransferase TrmD
Rv3054c		1630.1±1187.7	Conserved hypothetical protein
Rv3056	*dinP*	4.7±0.4	Possible DNA-damage-inducible protein
Rv3117	*cysA3*	6.6±1.6	Probable thiosulfate sulfurtransferase CysA3
**Rv3206c**	*moeB1*	61.7±7.9	Probable molybdenum cofactor biosynthesis protein MoeB1
**Rv3223c**	*sigH*	72.3±37.6	Alternative RNA polymerase sigma factor
Rv3279c	*birA*	6.4±2.4	Possible bifunctional protein BirA
Rv3280	*accD5*	2.1±0.5	Probable propionyl-CoA carboxylase beta chain 5 AccD5
Rv3347c	*PPE55*	42.4±11.5	PPE family protein PPE55
Rv3462c	*infA*	8.4±2.2	Probable translation initiation factor if-1 InfA
Rv3463		573.6±110.9	Probable F420 dependent oxidoreductase
**Rv3913**	*trxB2*	67.2±25.2	Probable thioredoxin reductase

^a^Rv numbers in bold were previously shown to have SigH-regulated expression in wild type compared to Δ*sigH* strain (11), or in a *sigH* overexpression strain compared to wild type (31), and to be directly regulated by SigH based on primer extension (11) or ChIP-Seq (31) data.

^b^Values are the ratio of expression of heat stressed/unstressed cells in wild type H37Rv divided by expression of heat stressed/unstressed cells in the Δ*sigH* strain.

Though over half of SigH binding sites in this study were linked to SigH-regulated transcription, a large proportion were not, indicating that a substantial number of the SigH binding sites that we identified do not function as SigH-regulated promoters under the heat stress condition we tested, where SigH is strongly expressed. Two potential reasons for this finding are i) off-target (non-promoter) SigH binding in the ChIP-Seq experiments, and ii) effects of additional regulatory factors, e.g. repressors or activators that may inhibit or enhance transcription from specific genes, and whose activity may be affected by different stresses. To address the first issue, we determined that expression of SigH-FLAG in response to aTc induction in the ChIP experiments was lower than expression of SigH from the native chromosomal gene following heat stress (**Fig 1B**), indicating that false positive binding sites are not likely to result from excessive induced expression of SigH-FLAG in these experiments. This result suggests that SigH binds to sequences in the *M*. *tuberculosis* chromosome in a manner that does not lead to transcription initiation in the conditions we tested.

To address the second issue, we selected 15 genes that did not show heat-induced increased expression, plus two positive controls, Rv0759c, which showed low-level SigH-dependent heat-induced expression, and Rv2466c, which showed high-level SigH-dependent heat-induced expression. The wild type and Δ*sigH* strains were grown to mid-log phase and subjected to heat stress or to oxidative stress with 50 μM plumbagin for 20 minutes. We verified increased *sigH* expression (17.3-fold) in response to plumbagin. The control genes Rv0759c and Rv2466c showed 2.4-fold and 118.9-fold induction, respectively, in response to plumbagin. Of the 15 genes that had not shown heat-induced increased expression, 7 showed low-level induced expression (2.3–3.6-fold), but none showed high-level (>5-fold) increased expression (**S4 Table).** The SigH binding sites linked to 2 of these genes, Rv0435c and Rv3596c (*clpC1*) contain sequences that match the SigH -10 and -35 consensus promoter sequences (see below and S4 Table)). MEME analysis did not identify an alternative consensus in the peaks linked to the other 5 genes that showed low-level plumbagin-induced expression. These data suggest that SigH targets the same promoters in response to oxidative and heat stresses, and that for the genes and conditions we tested, there are not stress-specific co-regulators that have major effects on gene expression.

### Intragenic binding sites

We identified 33 SigH binding sites that were wholly within annotated coding sequences, which we defined as intragenic. This proportion is similar to what was observed for the *E*. *coli* sigma factor FliA [34]. Several possibilities could allow for sigma factor binding within genes. These include regulation of a gene adjacent to the gene in which the binding site occurs, mis-annotation of translational start sites or coding sequences, sense or anti-sense transcription of non-coding RNAs, or binding that does not lead to transcription initiation. Our qRT-PCR results indicate that some intragenic sites are associated with SigH-dependent transcription, whereas others are not. Of the intragenic sites identified, 10 downstream sequences within the gene in which the binding site occurs showed SigH-dependent transcription following heat stress (>2-fold greater expression in wild type compared to Δ*sigH*), 4 were linked to SigH regulation of an adjacent gene and 19 were not associated with SigH-regulation of transcription in the same or an adjacent gene in the conditions we tested (**S1 Fig**). We also performed qRT-PCR targeting the region upstream of the binding site in a subset of genes in which intragenic binding sites were present in the 3’ half of the gene, to see if binding is associated with antisense transcription. Of the 8 genes analyzed, we were able to detect an anti-sense transcript in only one, Rv3056, which showed modestly (3.0-fold) increased heat stress-induced expression in wild type *M*. *tuberculosis* compared to the Δ*sigH* strain.

### Identification of the transcription start points of genes that show SigH-dependent transcription and determination of an optimal SigH consensus binding site

To precisely determine the sequence requirements for SigH-dependent transcription, we attempted to identify the transcriptional start point (TSP) for genes linked to a SigH binding site for which a 3’ gene showed at least 2-fold greater expression in wild type compared to the Δ*sigH* strain. Using 5’ RLM-RACE we successfully identified 25 TSPs that had not been previously identified. Of these, 18 were between 5 and 250 bp 5’ of the annotated initiation codon, 3 were within 5 bp of the initiation codon and 4 were >5 bp 3’ of the initiation codon. The first group of TSPs would produce transcripts with 5’-untranslated regions of RNA of various lengths consistent with typical leadered mRNAs, the second group are consistent with leaderless mRNAs but may also result from incorrect annotation of the initiation codon, while the third group suggests the possibility of alternative forms of the protein versus mis-annotation of the translational start site. All 7 previously identified TSPs are located in a position that would produce leadered mRNAs [11].

**Fig 4:**
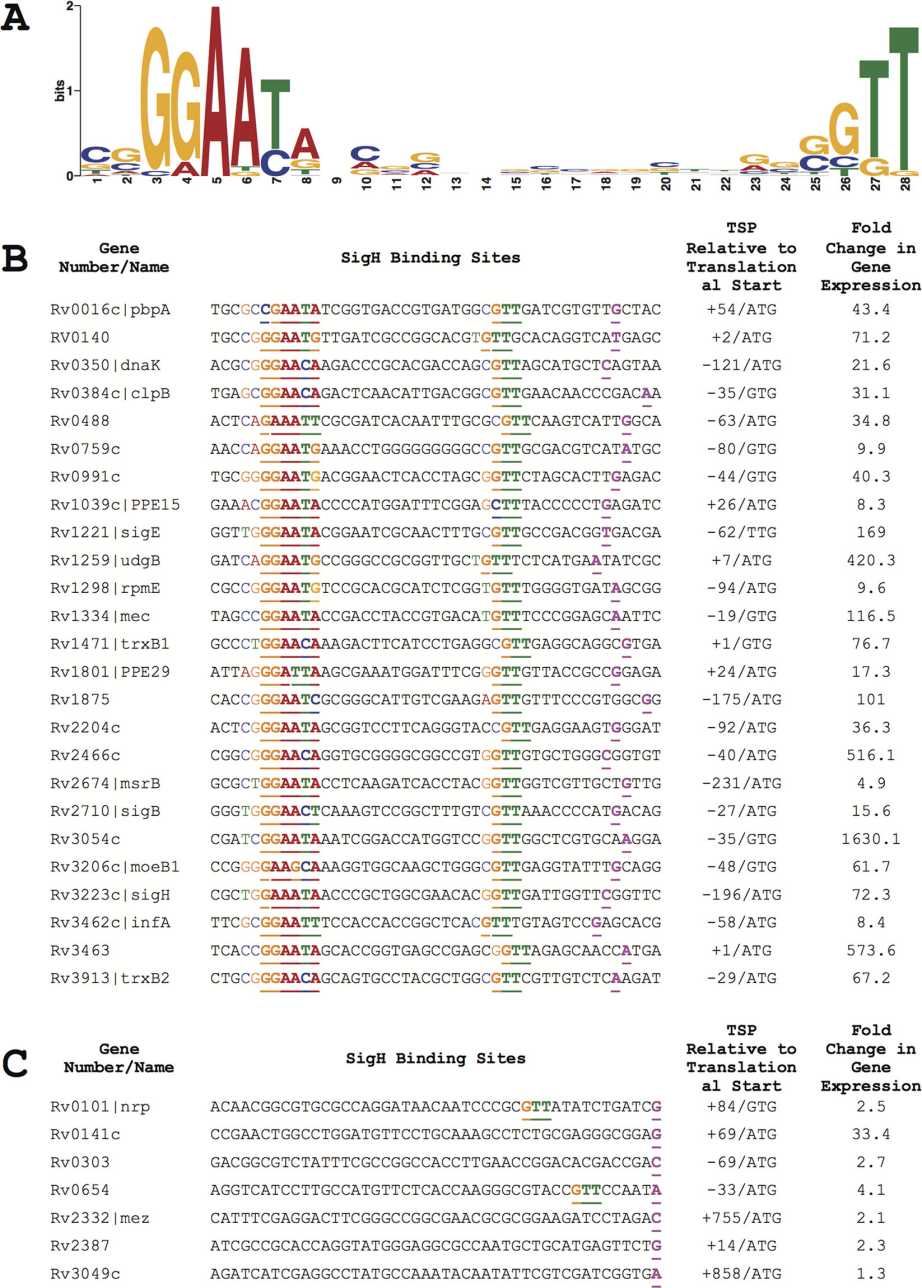
Sequence analysis of experimentally confirmed SigH promoters. 25 promoters experimentally identified in this research plus 7 previously identified promoters recognized by SigH were analyzed using the MEME analysis suite. **A**. LOGO showing consensus -10 and -35 promoter elements derived from these sequences. **B**. 25 promoter sequences that match the SigH consensus sequences. The position of the TSP and the ratio of heat stress-induced expression in wild type relative to expression in a Δ*sigH* strain are also shown. **C**. 7 promoter sequences that do not match the SigH consensus.

Using the MEME suite of software, we analyzed the 40 base pairs 5’ of these TSPs, together with the TSPs of the 7 promoters previously shown to be directly regulated by SigH [11], to identify conserved sequences. This analysis yielded a very strong promoter consensus of GGAAYR-(N_17_)-GTT (where Y is C or T and R is A or G) (**Fig 4**). 25 of these promoters, including 18 newly identified in this work, have sequences that match both the -10 and -35 consensus sequence elements with appropriate spacing between these sequences. The remaining 7 promoters newly identified in this work do not match the consensus **(Fig 4)**. In some cases, partial sequences corresponding to the -10 or -35 consensus are present at the appropriate position relative to the transcription start point. In addition to the sequence requirements for SigH binding indicated by these data, a key feature of the consensus is the restricted spacing between the -10 trimer and -35 hexamer. Of the 25 experimentally identified promoters, in 19 there are 17 bases between the -35 hexamer and the -10 trimer. In two promoters there are bases and in 4 promoters there are 18 bases separating these promoter elements.

Comparison of SigH-dependent transcription following heat stress of the experimentally validated promoters that contain the both the -10 and -35 consensus promoter sequences and those that do not, shows a striking difference between these groups. The genes 3’ of the 25 promoters that match the consensus show a mean 173-fold SigH-dependent induction following heat stress, with only 1 showing less than 8-fold induction (**Fig 4**). Among the 8 promoters that do not match the consensus sequences, the median induction is 7.1 fold with only 3 showing greater than 3-fold induction. Similarly, the median binding enrichment in the ChIP-Seq experiments was lower at sites containing promoters that lacked both the consensus promoter sequences (11.6-fold) compared to sites that do match both of these sequences (18.6-fold).

## Discussion

In this work we comprehensively identified SigH binding sites throughout the *M*. *tuberculosis* genome. We then determined whether these binding sites were associated with SigH-regulated stress-induced transcription, and for those that showed increased expression in wild type compared a Δ*sigH* strain, we attempted to identify the transcriptional start point and evaluate candidate promoter sequences 5’ of these genes. Based on the presence of a SigH binding site, SigH-dependent stress-induced transcription and promoter sequences that match the consensus sequences recognized by SigH, we identified for the first time at least 18 genes that are directly regulated by SigH, increasing the direct SigH regulon by over 3-fold. Though some of these genes were previously shown to be differentially expressed in wild type *M*. *tuberculosis* compared to a Δ*sigH* strain, we also identified several genes that are regulated by SigH that were not previously suspected of being part of the SigH regulon. Though our genome-wide approach with follow-up validation has allowed us to markedly expand the number of genes directly regulated by SigH, these genes may not comprise the entire SigH regulon. For example, *clpB*, which has been shown to be negatively regulated by the HspR heat shock repressor that binds to a site that overlaps with the SigH-regulated promoter [11, 30] was not identified in our ChIP-Seq experiments. Other genes that are co-regulated by transcriptional repressors that occlude SigH binding sites may not have been identified under the conditions we used. For genes linked to SigH binding sites that show SigH-dependent stress-induced expression, it is likely that our 5’-RACE experiments did not detect some SigH-regulated promoters.

The newly identified SigH-regulated genes markedly expand the functional roles of SigH-regulated gene expression in response to stress. Prior results had demonstrated that SigH is important for *M*. *tuberculosis* to re-establish redox homeostasis following oxidative stress, through regulation of several components of the thioredoxin/thioredoxin reductase system, as well as other oxidoreductases, and for repair or removal of damaged proteins by the SigH-dependent induction of several heat shock proteins/chaperones [11, 12]. Our findings in this work indicate a broader role for direct regulation by SigH in recovery from oxidative and other stresses. Direct SigH regulation of the DNA repair gene *udgB* for example, a gene that was not previously suspected to be SigH-regulated, indicates a role for SigH in recovery of genome integrity following genotoxic stress. SigH regulation of *rpmE*, which encodes a zinc-binding ribosomal protein gene indicates a role for SigH in recovery of translation by replenishment of damaged ribosomal proteins. The orthologue of this gene was shown to be regulated in *Streptomyces coelicolor* by the SigH orthologue SigR [35], Also striking is the direct role of SigH in regulating sulfur metabolism in the mycobacterial cell, including control of genes predicted to be involved in sulfur transport and incorporation of sulfur into molybdopterin. A critical role SigH appears to be the synthesis and salvage of cysteine-containing proteins, which are particularly susceptible to oxidative stress. Our data show direct SigH regulation of genes encoding an alternative cysteine biosynthetic pathway encoded by the operon Rv1334-Rv1336 (*mec*, *cysO* and *cysM*) and Rv3206c (*moeZ* or *moeB1*) [36]. Further demonstrating a role for SigH in replenishing sulfur-containing amino acids, we found that SigH also directly regulates *msrB* (Rv2674), which encodes an enzyme that repairs oxidized methionine residues.

Though 41 of 69 of SigH-binding sites were linked to SigH-dependent gene expression, the expression of genes adjacent to many binding sites did not show evidence of SigH-regulation in response to heat stress. Testing the expression of several of these genes in response to plumbagin-induced oxidative stress showed low level SigH-dependent expression for 7, however most did not show increased expression in response to either stress. The lack of gene regulation linked to a subset of binding sites of sigma factors or transcription factors has been a consistent observation in many ChIP-Seq studies [31–34]. In some cases these may be regulatory sites where transcription of the gene of interest is co-regulated by other transcription factors so that changes in gene expression were not observed under the conditions tested. For other binding sites that we identified in the ChIP-Seq experiments where transcription regulation is not evident, it is likely that binding does not indicate a site at which SigH regulates gene expression. Transcription initiation is a multi-step process that requires binding of RNA polymerase via sigma-promoter interactions, DNA melting and initiation of elongation, which is frequently aborted without the RNA polymerase being able to escape the promoter to transcribe full-length mRNAs [37, 38]. Thus, some sites to which SigH binds weakly may not achieve the initial steps required for transcription initiation. This inference is supported by the markedly greater frequency and magnitude of SigH-dependent transcription following heat stress from binding sites that match the SigH promoter consensus sequences compared to those that do not. Conversely sites to which SigH binds strongly that are not linked to regulated transcription may have sequence or structural characteristics that do not allow efficient completion of the steps required for transcription initiation.

In previous work, based on a small number of genes directly regulated by SigH, we identified a SigH binding consensus and subsequently performed extensive mutagenesis of the -10 and -35 sequences [11, 19]. This research identified optimal promoter sequences for SigH and for SigE, and identified position 6 of the -35 hexamer as important for distinguishing SigH from SigE dependent promoters, though some promoters are recognized by both. The much larger number of SigH-regulated promoters experimentally identified in this study, derived from SigH-controlled genes identified using an unbiased genome-wide methodology, has allowed us to define the optimal and tolerated SigH promoter sequences more precisely. Notably, while our results show that the optimal consensus is GGAAYR-(N_17-18_)-GTT, it is clear that variation that was not previously known can be tolerated at the 2^nd^, 5^th^ and 6^th^ positions of the -35 hexamer and at each position in the -10 trimer. In addition, while our previous results showed that SigE prefers a pyrimidine at the 6^th^ position of the -35 hexamer [19], results from this study show that SigH strongly prefers a purine at this position.

In this work we have identified a greatly expanded direct SigH regulon that markedly broadens the role of SigH in stress response and recovery. In particular newly identified SigH-regulated genes indicate a direct role for SigH in DNA repair, sulfur metabolism, synthesis and salvage of sulfur-containing amino acids and recovery of translation. In addition to these specific new insights into SigH function, this research demonstrates the value of genome-wide approaches to understanding bacterial gene regulation, while highlighting the importance of targeted follow-up experiments to more fully understand the implications of the genome level data.

## Supporting Information

S1 Fig Map of genomic loci containing SigH binding sites identified by ChIP-Seq. For each SigH binding site, the region containing a binding site is shown. Black bars under each locus indicate the location of the binding site. The expression ratio (fold induction following heat stress in wild type divided by fold induction following heat stress in the Δ*sigH* strain) is shown for genes adjacent to each binding site. Where an expression ratio >2 was observed, the arrow is black; where an expression ratio is <2 the arrow is grey. Arrows corresponding to genes that were not tested are white.
(PDF)

S1 Table Primers used for qRT-PCR. (DOCX)

S2 Table Primers used for 5’-RACE. (PDF)

S3 Table ChIP-Seq binding data. (XLSX)

S4 Table Gene expression following oxidative stress. (DOCX)
