# Supplementary figures and images for: Comprehensive Definition of the SigH Regulon of Mycobacterium tuberculosis Reveals Transcriptional Control of Diverse Stress Responses
Source: PLoS One. 2016 Mar 22;11(3):e0152145. doi: 10.1371/journal.pone.0152145 (PMC4803200; doi:10.1371/journal.pone.0152145)

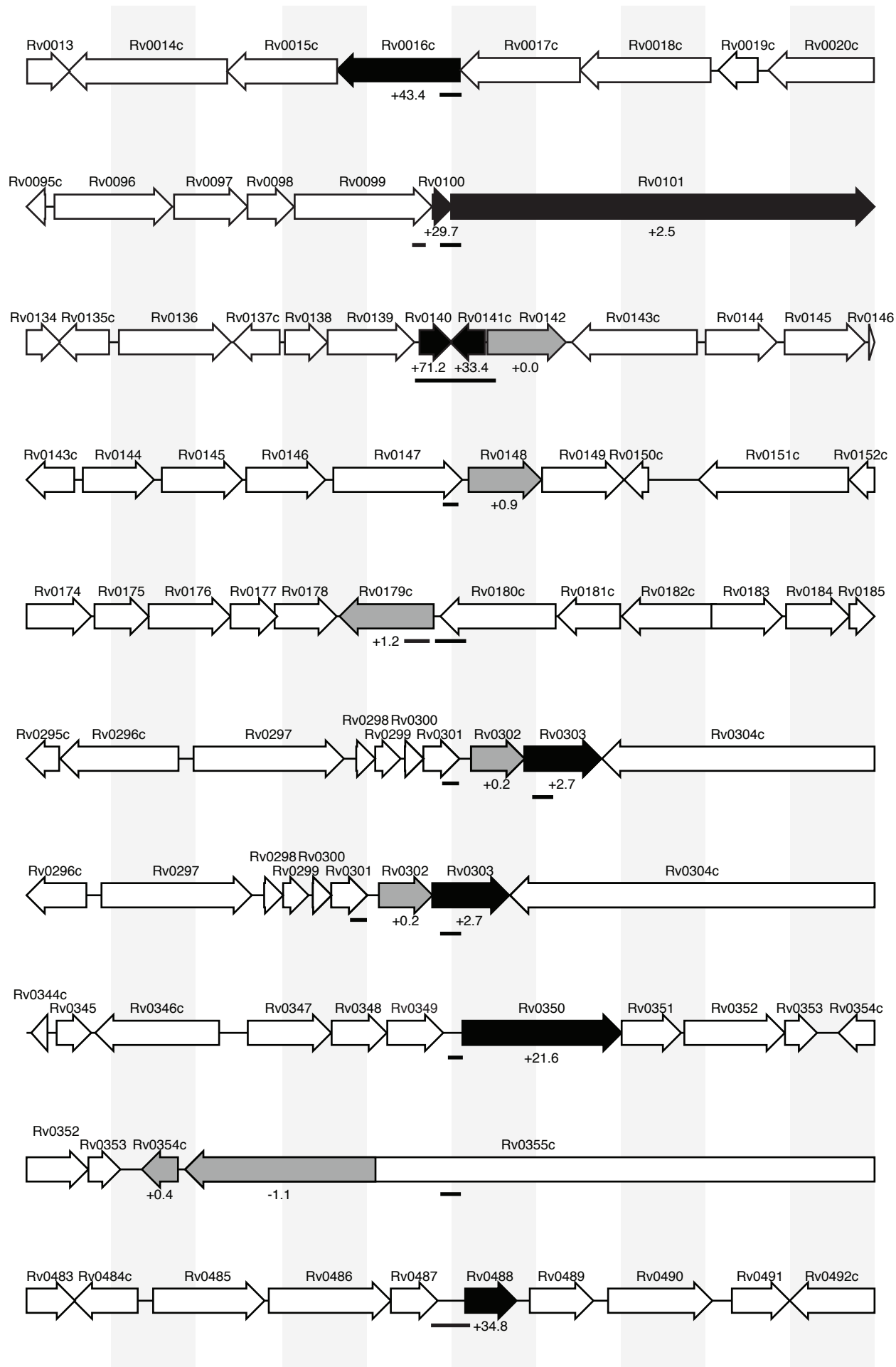

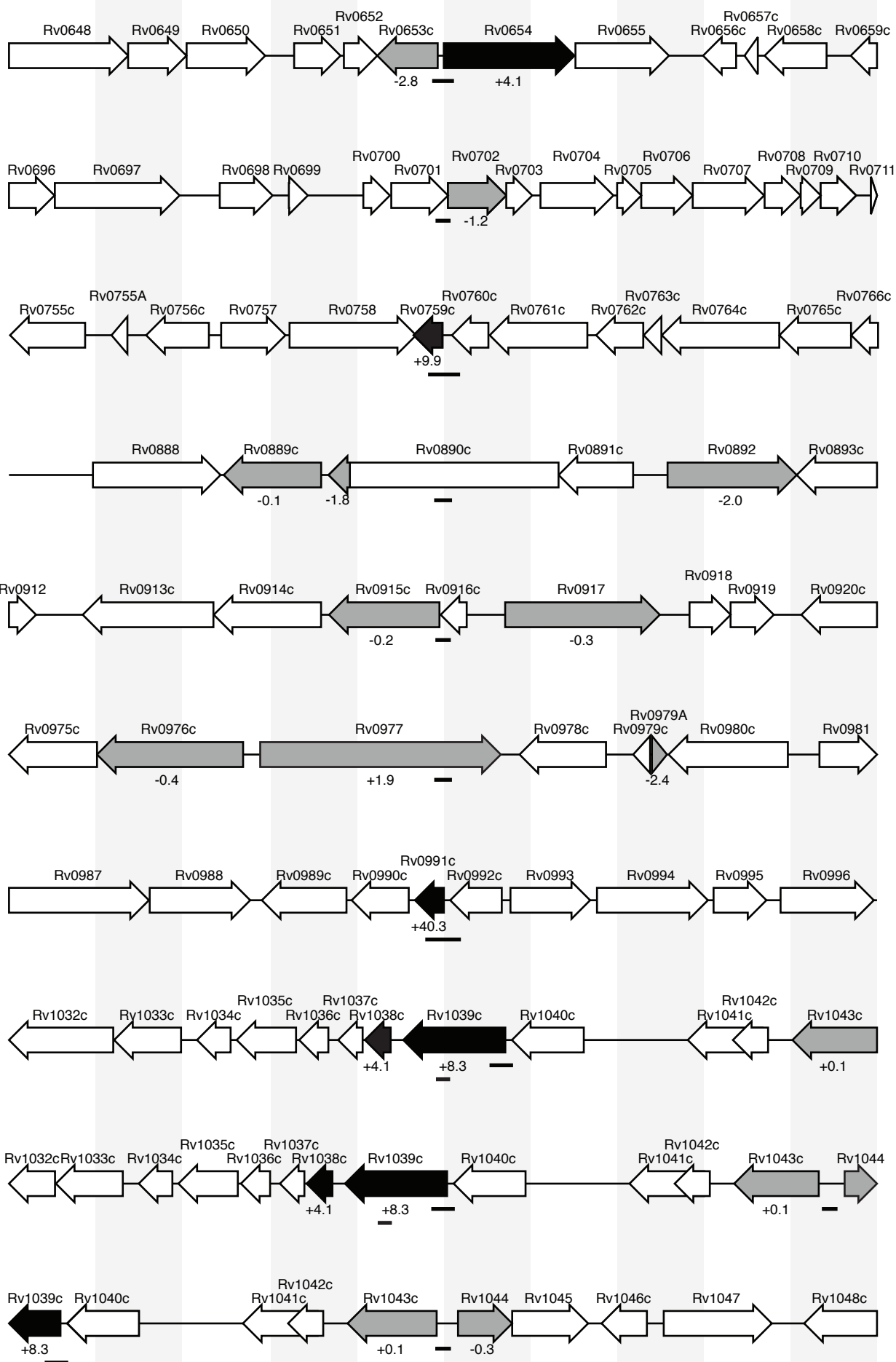

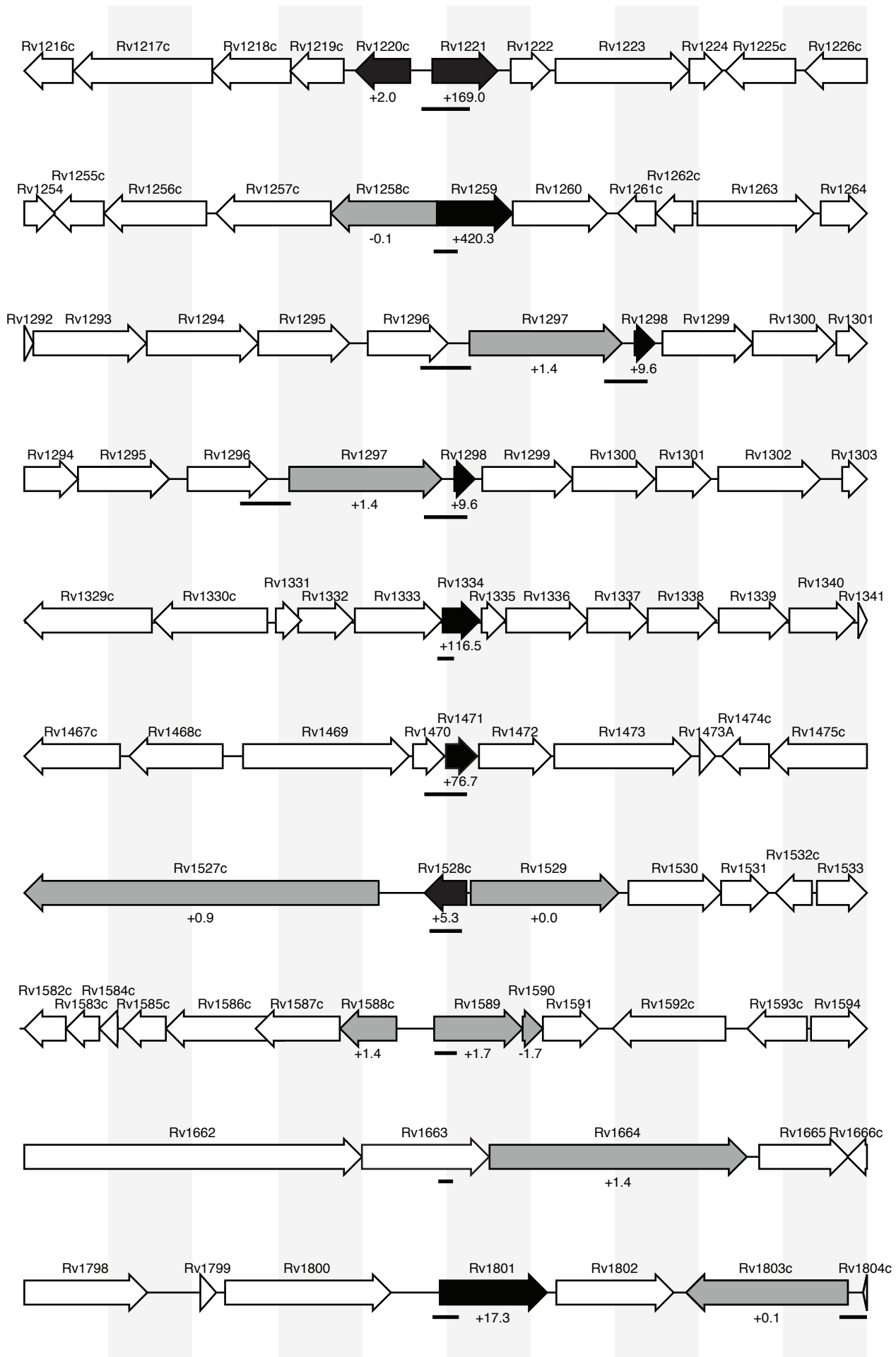

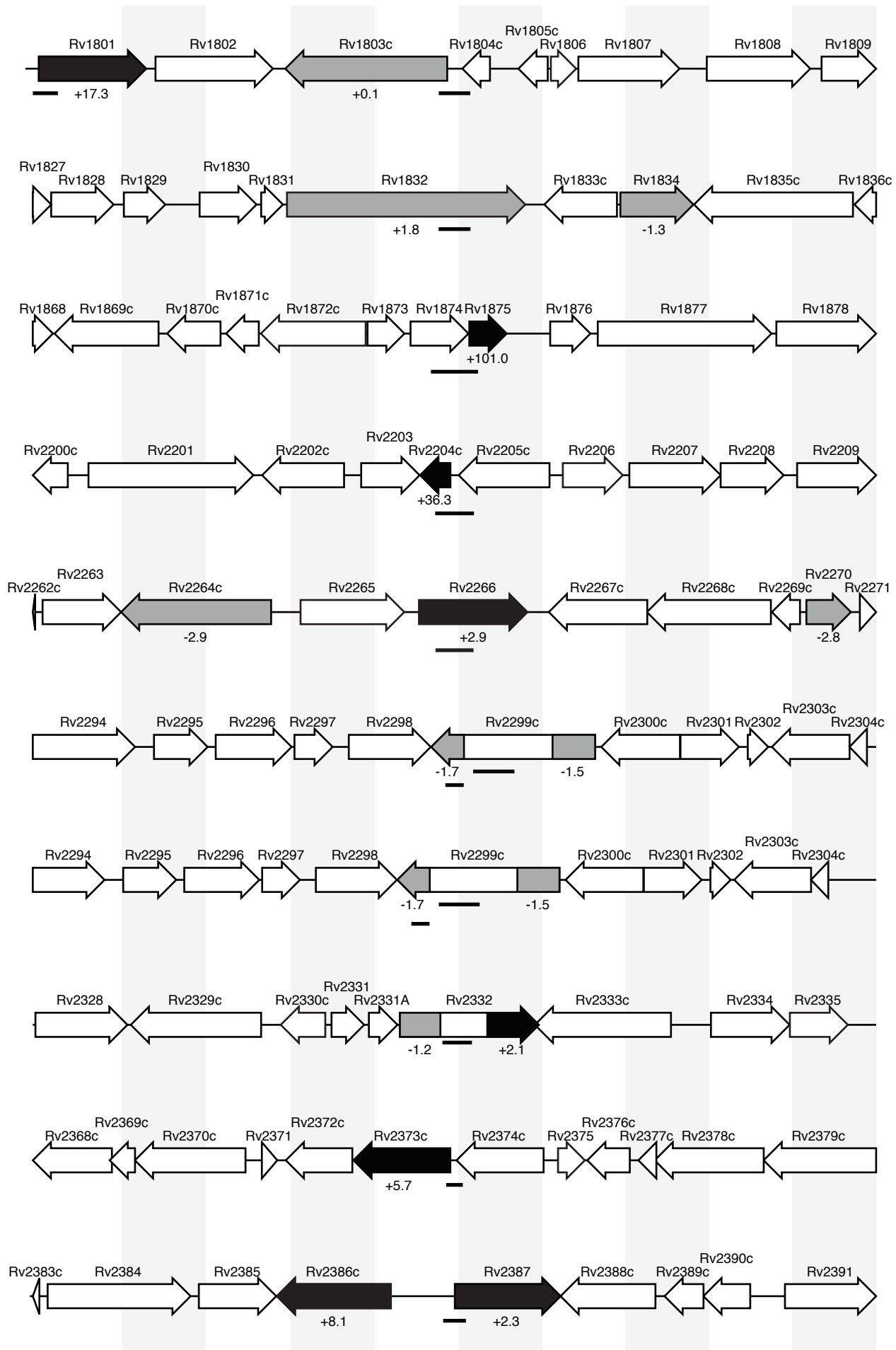

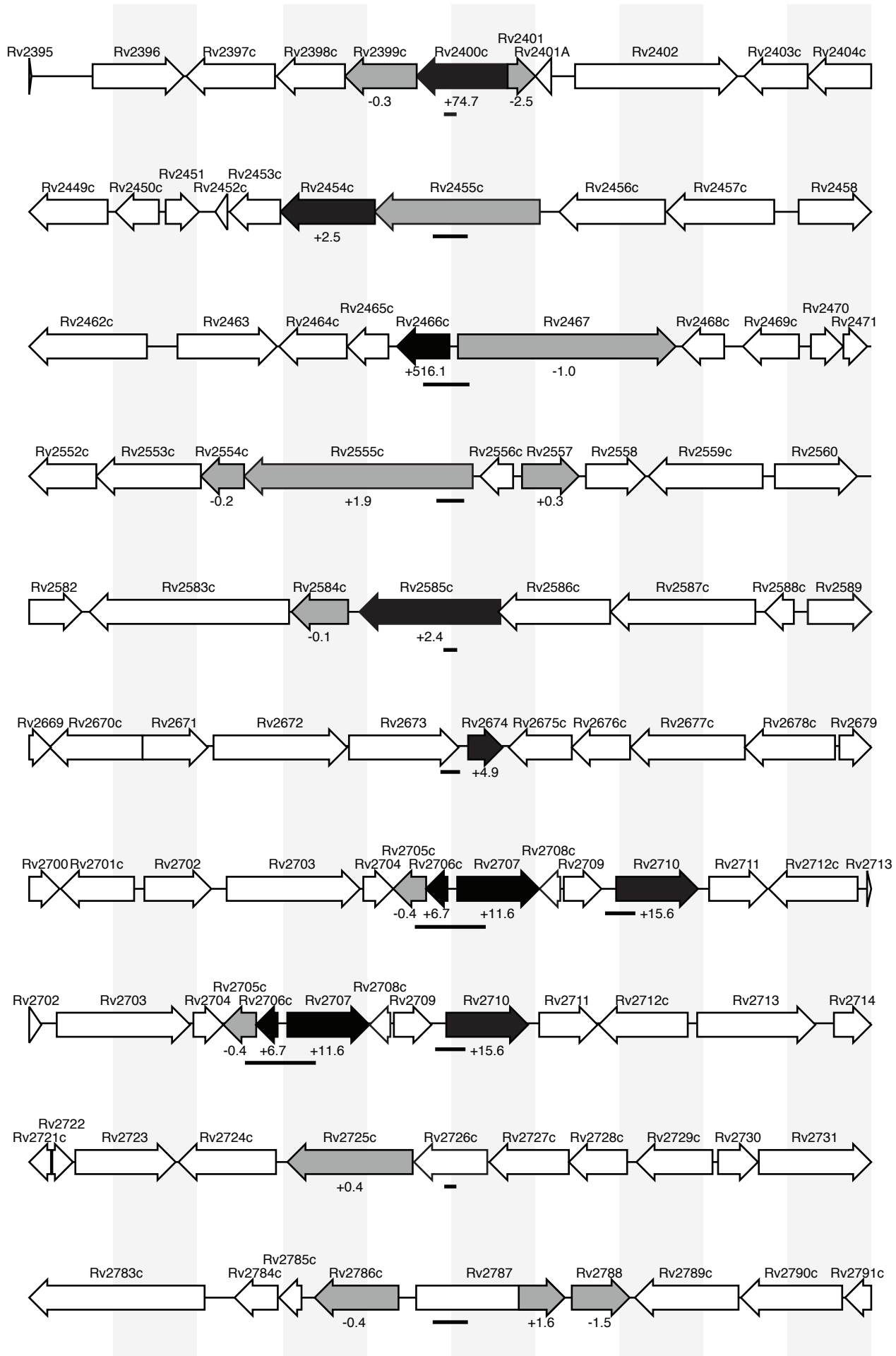

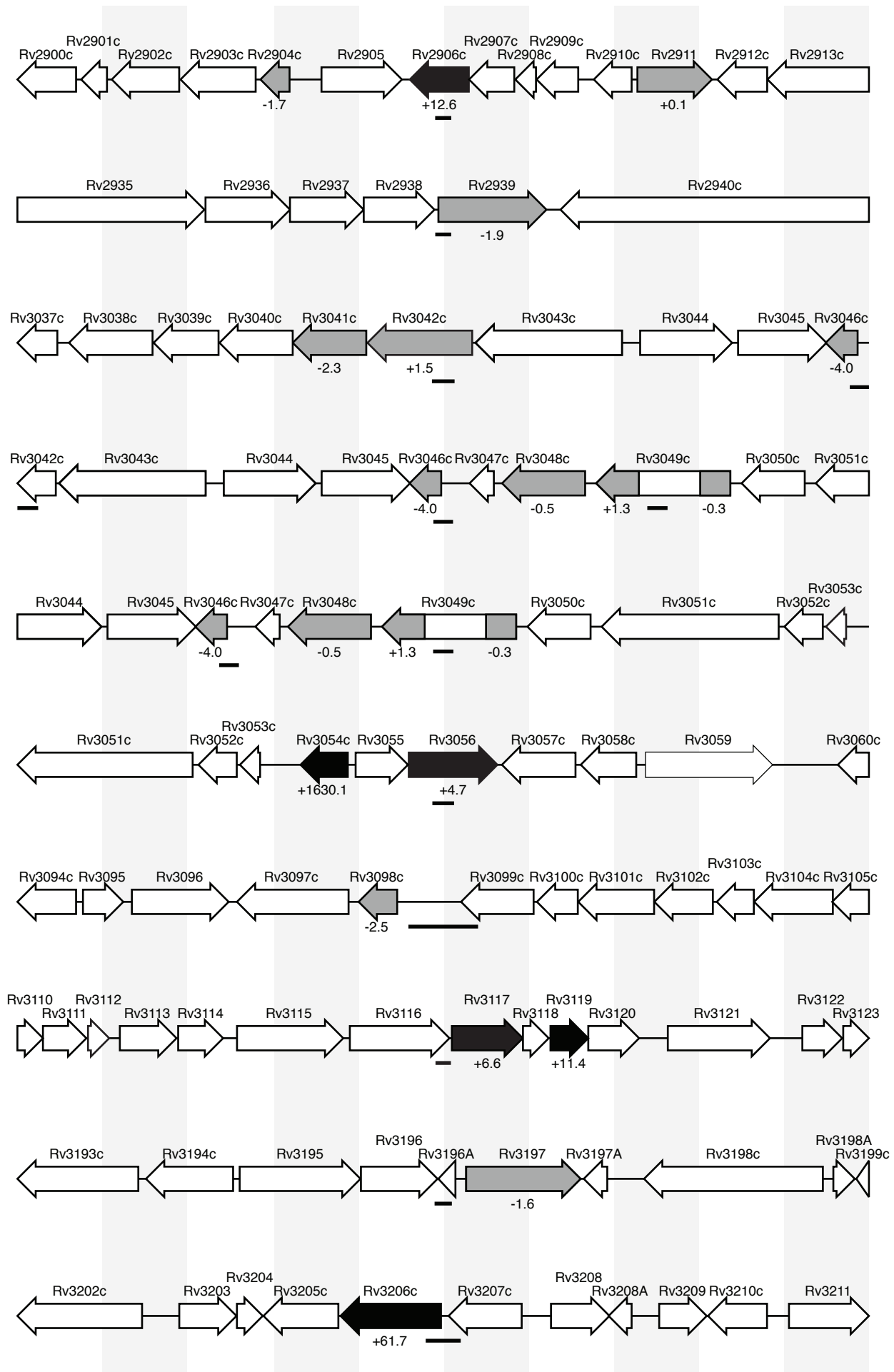

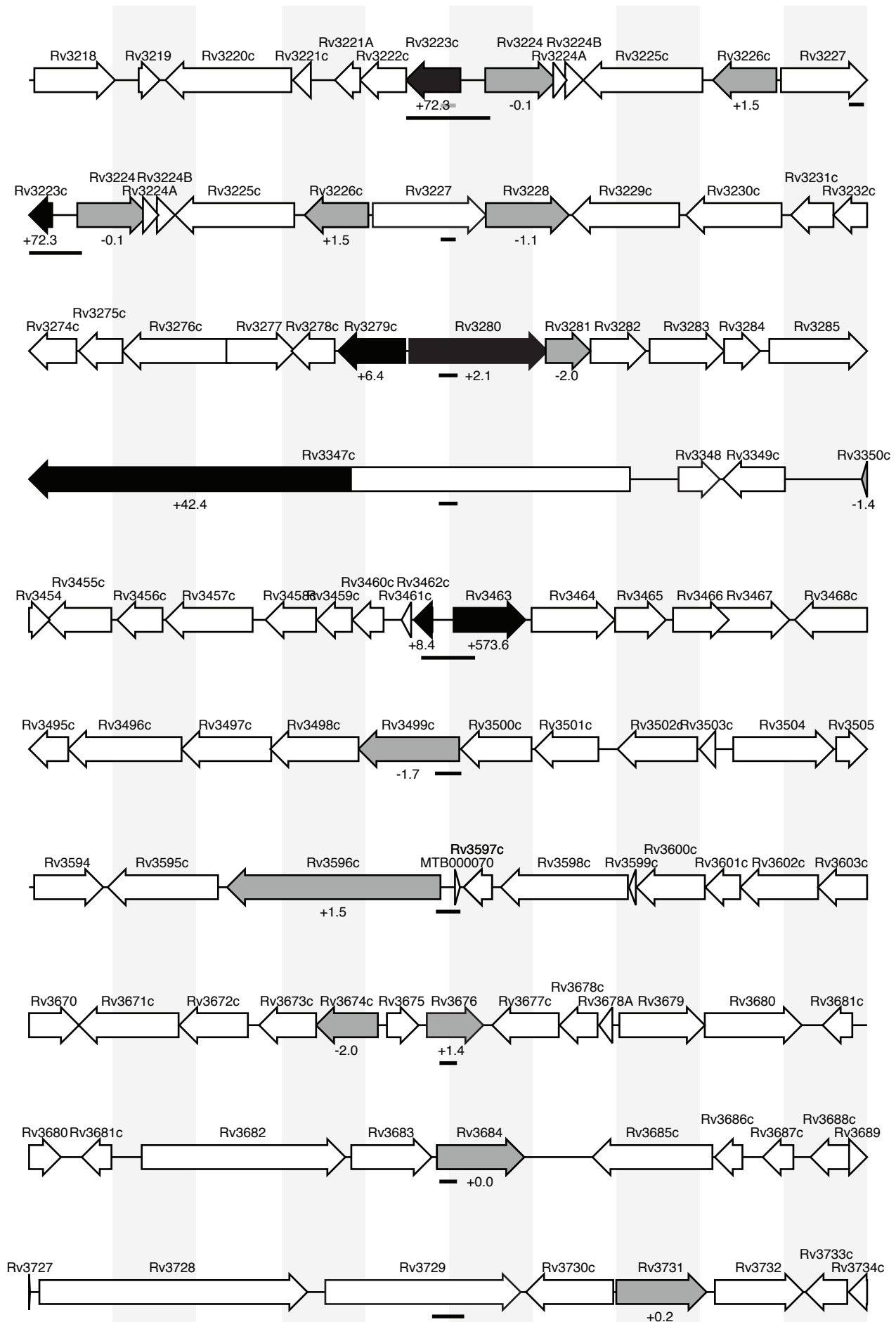

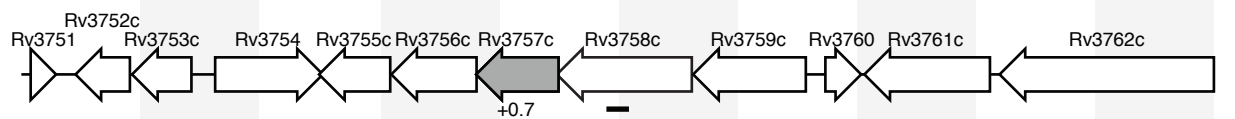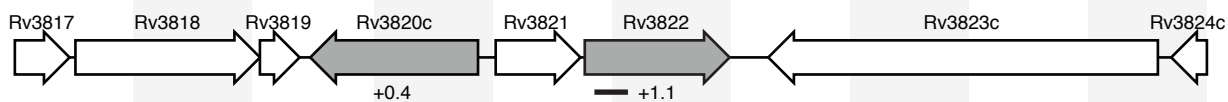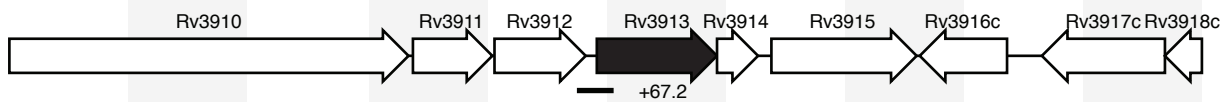

Supplement: S1 Fig — For each SigH binding site, the region containing a binding site is shown. Black bars under each locus indicate the location of the binding site. The expression ratio (fold induction following heat stress in wild type divided by fold induction following heat stress in the ΔsigH strain) is shown for genes adjacent to each binding site. Where an expression ratio >2 was observed, the arrow is black; where an expression ratio is <2 the arrow is grey. Arrows corresponding to genes that were not tested are white. (PDF) [file pone.0152145.s001.pdf]
