# Supplementary material for: Comprehensive Definition of the SigH Regulon of Mycobacterium tuberculosis Reveals Transcriptional Control of Diverse Stress Responses
Source: PLoS One. 2016 Mar 22;11(3):e0152145. doi: 10.1371/journal.pone.0152145 (PMC4803200; doi:10.1371/journal.pone.0152145)
Supplement: S1 Table — (DOCX) [file pone.0152145.s002.docx]

**Table S1.** Primers used for qRT-PCR

| **Primer used for qRT-PCR** | | |
| --- | --- | --- |
| **Rv No.** | **Forward Primer** | **Reverse Primer** |
| Rv0016c | TCGTTTCCTGCGGGTCTATC | TCGGACCCGTTCAATATCGG |
| Rv0100 | TGTTGTATATCGACGAGGCGG | GTTCGGATTGTCGGTTCACG |
| Rv0101 | ACCAGCTTCATGGTGGTAGC | AGCCCACCAAGTTATCCAGC |
| Rv0140 | CGACGTGGTACAGGATAGGC | TACGTCCACATCACGTCGTC |
| Rv0141c | CCCTCGACCACTACCGTTTC | CTACTGGAGCATCGTCACCC |
| Rv0148 | TTCCGTGAGCAGAGTTACGG | AGCGTATTGATCAGGCCGAC |
| Rv0179c | GGTAGATCTCGGAGGCTTGC | TCAAGAATGCCTCCCAACGG |
| Rv0302 | ATACCCACCTGGGATGAGGC | TGACTTCGTTCCATCGACAGC |
| Rv0303 | TCGATACACCACGTCCAAGC | CGGAGCCCGGCATTAGG |
| Rv0350 | GTTCGAGTCAGTCCACCTCG | CAGCAGGTCCTGAGTGATCC |
| Rv0435c | AAGTGAGCATCTCGAGTCCG | GCGCCCAAGGTCTGTAGTAG |
| Rv0488 | GATCTGCGGTGGTTTTTCGG | ATCAAGAATGCGCCAAGCAG |
| Rv0653c | TGTACCGGCTAATGTTCGGC | GCACCACATGCGCGAAAC |
| Rv0654 | GTGTCCTACTCGTTCGCCC | CGTAGTTGTCGGTCAGGGAG |
| Rv0702 | TGGAAGGTCAAAACCCGTCG | GCAGATTGCGCACGCTTTTC |
| Rv0711 | AAGAGTTCACCGACGGGTTC | ATTCTTCGAGTGGCGTCAGG |
| Rv0759c | GTGGATCCCGCGTTATTTGG | ATGTGTAGGTGGGGCACTTC |
| Rv0889c | TGTCCTATGTCGCGCAATCC | AATGGCCTCGATGTGTCTGG |
| Rv0915c | GACATTCTGTCCGCATTGGC | CAATTCCCCTATCGGGGTGG |
| Rv0991c | GCAAGCTGTTCAATGCGGTC | AACTCTTCGTCGACTCGCTG |
| Rv1038c | AGTCATAACCTGTCCGCCAC | GCGGATCCGTCATAAAACGC |
| Rv1039c | AACACACCGGCAATCATGG | CGGGCTTAACGGTTGCAG |
| Rv1043c | GGGCGATATCGAAGGGACAG | ACTATCCACCGGATTGCCAG |
| Rv1221 | TCGGTCCAGAATTACCAGCC | GGTCGTAGTCCTCGGGTAAC |
| Rv1259 | TATCGCGGCTGAATCTAGCG | CTCTACCGGGGACCTGAACG |
| Rv1297 | TCTGGAAACCAGTACCGAGC | CTGCAGGATCGTTGTCTTGC |
| Rv1298 | ATACCTTCCAGACGCGTAGC | CTTCTCGAAGCGAGCCACC |
| Rv1334 | TATCCCGATGACCAATGCCG | TCGCAGTGTGCGAGTGATAG |
| Rv1471 | CGAGTCGTCGGAAAAACACC | TGGTTGAACAACAACTTGCCG |
| Rv1528c | GTTGGATCCTCTAGGTGGCG | ATCCAGCCATCGCCGAATAC |
| Rv1801 | ACACCTTTCCCGGGGTATTG | CTCCGGTGATTGGTTTCCCC |
| Rv1803c | CGGACCGATTAGTGTCCAGG | CGTATAGCACCCCACCTTGG |
| Rv1875 | GTGGTTTCCACCGTTCGTG | GGTTGCCGAGTTTGACCTTG |
| Rv2204c | CGCTGGATTGCGCTATAACC | TCGACGAAATCGATCGACGC |
| Rv2226 | ACGAGTCAAAGTTCGCCGAT | TTGCGTGCTAGGTCGAATGT |
| Rv2332 | TTCTGAGCATCGACGAACCC | CACACCCCAGTCACCGATAC |
| Rv2373c | GACGTCTTTGTCCGCGAAGG | AATGGTGATCTCGCTCAGGC |
| Rv2386c | CATTTCAGTGCGCTCTTCGC | ATGGATCCAACCGTGCTCTG |
| Rv2387 | GCACGAATGCGTCGATCTTC | GACCTTCTGTCCCTGCAGTC |
| Rv2400 | TCAGCTACGAGAACGAAGCC | CTCCAAGGTGTGTGCTGGTC |
| Rv2454c | ATCAGCGGTATCGGATGCTC | CCATACCGACAGATCCTCGC |
| Rv2466c | TTCTGGTTCGATCCGCTGTG | GGTCGTCACGGTTTTCGTTG |
| Rv2585c | TGGATGCCTATGACTTGCAC | TCACCGCATACATTTTCGTC |
| Rv2674 | CTGTGGCGCCGAATTGTTC | GTCATCCCCAACGAGTGGTC |
| Rv2706c | GCAGAAAAGAAGCTCGGCTC | AGAACGGAGAGCCATGAACG |
| Rv2707 | GGTGTTCCTAGTAGCGACCG | GATGACCCCGACGGTTAGAC |
| Rv2710 | ACCTGCTGGAAGCAAACCTG | TCGAACTTCTCCATCGCTCG |
| Rv2906 | CGAAGAAGTCTCGATCGGCG | GAGTGCGAATCATCTTGGTGG |
| Rv3049c | CGCACCTGTACTCCTTCTCG | TTGAACTCGATGTAGCGGCG |
| Rv3054c | TTCAACCGCCAGATCGCC | CACCTCCGTCGCTGTGTC |
| Rv3056 | ATCAGTGACAACAAGCAGCG | TGCCAGCCTTTTCGTAGTCT |
| Rv3117 | CCTTCAAGTCCGATGAGGAG | TTGGTGTCCGAGTAATTCCC |
| Rv3119 | ATTTGTGGGAAACGTGCGTG | CCTTGTCTTCACACCGTCCG |
| Rv3206c | TGGGCATTATCTGTGCCTCG | TGCGATAGCTCATCTCCAGC |
| Rv3223c | GACATCGATGGTGTAACCGGTTCGG | TCACCATCGTCTCCTGGAGCAAGTC |
| Rv3279c | TCAAGTGGCCCAATGACGTG | ATCAACCTCTTCGGGGGC |
| Rv3280 | CCTATGACATGCACGAGGTG | ATGCCGTTGTATTCCTGGTC |
| Rv3347c | TCACCGTCTCAAACGTCCAG | GTAAGGGGTATGGCGACCTG |
| Rv3462c | CCATGTTCCGCATTGAGCTG | TCGTAGGGCGACAATTCCAC |
| Rv3463 | AATCGTTCCACCGCATCGAG | GCCGGTCTAGGTATTCCACC |
| Rv3596c | ACCTACGCGAGTTCGATGAG | TGTGCGACCAGTGTCTTCTC |
| Rv3731 | CCTATCAACCGGACAAACGC | TGGTAAAGCCCTAGCAGCAG |
| Rv3757c | ATTCGCGGATCGCTCAGATG | CTATCACCGAACCCACGGAC |
| Rv3820c | TTCGGCATACCATCGCTGAC | TCCCGAACAAGAAGCAGTCC |
| Rv3913 | ATCAGGACATCGCCGTCATC | AGCATGATTTTGGAAGCCCG |
