# Supplementary material for: Comprehensive Definition of the SigH Regulon of Mycobacterium tuberculosis Reveals Transcriptional Control of Diverse Stress Responses
Source: PLoS One. 2016 Mar 22;11(3):e0152145. doi: 10.1371/journal.pone.0152145 (PMC4803200; doi:10.1371/journal.pone.0152145)
Supplement: S2 Table — (PDF) [file pone.0152145.s003.pdf]

Table S2. Primers used for 5' RACE

**Adapter**	GCUGAUGGCGAUGAAUGAACACUGCGUUUGCUGGCUUUGAUGAAA	
**Outer forward**	GCTGATGGCGATGAATGAACACTG	
**Inner forward**	CGCGGATCCGAACACTGCGTTTGCTGGCTTTGATG	
**Rv No.**	**GSP1 (Outer PCR Reverse)**	**GSP2 (Inner PCR Reverse)**
Rv0016c	GCTACCGAGTACGCCAGCAGTTG	GCTGCCGTGAATACTCGTCGAGCAACACTC
Rv0100	CCGCCTCGTCGATATACAACACGTC	CCACGAACGACCACTCTGGCCTTGTCTG
Rv0101	CTGCAGGTGCTCATCGGCTGAC	CGGATAGTCTGCCCCCGAATTCTCCAGGAC
Rv0140	CACCTCGCCATTGACGCGTACC	GTCGGCTCGATGGTGATCGGGTGATCG
Rv0141c	TCTTGTCCAATTCCGTACGGGTGAC	TGCTCCAGTAGGTGAAGTCGTTGCTGAG
Rv0303	GTCGTCGGTGAACGCGATGC	GCATTGCACACCAGGGCACGAATCGG
Rv0350	CGAGCCATGGTGAATCCTCCTGAATATG	CACTATGATGAGTGCACCCCGCTCAAG
Rv0488	AATCAGTGCCCCATCAGCGATCC	GACCAGCACGTATTCTCGCCTAATTCCTTGGC
Rv0654	GATAACGCCCGTCGAGGTGTTCC	CGCCAGGAAGCCCTCGAGATATGGGTTTTG
Rv0759c	GTCATCGGCTCAATCGTCAAGAACG	CCGGGCAGTTCACGGTTGATGATCTTGGTG
Rv0991c	GTAGAAGCCGGTGCCTTTGAACACC	TGAACAGCTTGCGCAGCCGGCCAGAACACC
Rv1038c	GCTAGGAATATCGCGGTGGGGATG	CTGCGCTGTTGATGATGTTTGCCACGAACG
Rv1039c	GTCATCGCATAGGCCGCCTCGTAG	CGCAGCGGCTTCGGCGGTGTAG
Rv1259	CGTTGAGTTCGCAGATCGAG	GTCTGCGGTGTCGCCGGATCAC
Rv1298	ATTGCCGCATCCGCAGACCACGGTGG	AGCATCGATTATGCCAGGTCAACCG
Rv1334	CAGCACTCCGCAGGCTTCGTC	ATGGGCCACCATCGCATTCACCAGGTC
Rv1471	GTCGGGGTGTTTTTCCGACGACTC	TAATCGACGAGCACCATGTCGCTGCTTTGG
Rv1801	CAAAGCCGATAGCTCCGAGGCGTAG	GTAGCCAGCCGCTGTTGCGTGCAG
Rv1875	TCGATGTTCTGGTTGGCACCGAATG	CGGCTTATCCGATTCCGCAGATCGTTGTCG
Rv2204c	TGGGGAAAATCCCACTTCCTCAACG	GGTACCCTGAAGGACCGCTATTCCCGAGTC
Rv2332	CTGTATACGGCGGCCAAGACCAC	TCGTTGAACACGCAGTAATCCGTGCCGTATG
Rv2373c	GTGCCACGCTTGATTCTCCTATGCC	CTCCTATGCCGCGTCTTTATGCCGCTTCTC
Rv2387	CGGCTTGATCTTGGCGAGTTCTTCG	GCAGCAGATACAGGGTTAGGCCCTGGTAGAG
RV2466c	AGTTCACCTCGATGTCGCGGACCT	GCGGATCGAACCAGAAATCGGCGACAGAC
Rv2674	CGTCATGGGTCCACGCTAGTCC	TGGATCGATACCGCCGTCCAGCCGGTTGTC
Rv2706c	AGTCCTGAGCTGACTAGAGCAGACC	CCCTCCGGAGCCGAGCTTCTTTTCTGC
Rv2707	AAGATCGAGTCGTCCCAGCTTTTGG	GTCGTCCCAGCTTTTGGACAAAGTCCTACGG
Rv3049c	CACCGCGGGGTAATAGGTAGAGGAG	GAGGAGTTCAGGATCCGTTTGCAGCCGATG
Rv3054c	GACGGTGACGCCGTCCGGAGCGAC	ACGATGCCCTGAGGCCACCTTCTTGC
Rv3119	GGTCATATGCACTGGTGTCCTCGTC	CTCGTCCACTTCGACGAAAACGACCTTCGG
Rv3206c	CCGTCAACACCCAGGTCAGGAATG	GTCAGGAATGATGAGATGGCGGCTGTAGCG
Rv3279c	GGATGAGGATTCGTTCGTCGAGCTG	GCCAAACACCGCAGCACCAACTTCAATCTC
Rv3347c	GCCCAAACCCACGTTCAAGTCG	CTGCCCACATCGGCCAACCCGAGATTGATG
Rv3462c	CGAATGTAGTGCTGACGCATCTTGC	TCTCCAGCTCAATGCGGAACATGGCATTGG
Rv3463	GTAGCCCAGCGCCTCGATTTCTGTG	GCGCCTCGATTTCTGTGGCCTGCTG
Rv3913	GGGACCGGAGCCGATAACGATCAC	CACGTCGCGAACGGGGTGGTGTG
