# Supplementary material for: Comprehensive Definition of the SigH Regulon of Mycobacterium tuberculosis Reveals Transcriptional Control of Diverse Stress Responses
Source: PLoS One. 2016 Mar 22;11(3):e0152145. doi: 10.1371/journal.pone.0152145 (PMC4803200; doi:10.1371/journal.pone.0152145)
Supplement: S4 Table — (DOCX) [file pone.0152145.s005.docx]

**Table S4. Gene expression following oxidative stress**

**A. Expression of genes linked to SigH-FLAG ChIP-Seq binding sites in response to 52^o^C heat stress or 50 μM plumbagin oxidative stress for 20 minutes**

| **Rv No.** | **Gene** | **H37Rv/Δ*sigH*** | | **Gene product** |
| --- | --- | --- | --- | --- |
|  |  | **Heat** | **PL** |  |
| 0148 |  | 0.8±0.5 | 0.7±0.1 | Unknown protein |
| 0179c | *lprO* | 1.8±1.3 | 1.9±2.0 | Possible lipoprotein LprO |
| 0302 |  | 1.3±0.3 | **3.2±2.9** | Probable transcriptional regulatory protein |
| 0435c |  | 1.8±0.3 | **3.1±2.1** | Putative conserved ATPase |
| 653c |  | 1.2±0.3 | 1.7±0.6 | Possible transcriptional regulatory protein |
| 0702 | *rplD* | 0.4±0.1 | 0.6±0.2 | 50S ribosomal protein L4 RplD |
| 0889c | *citA* | 1.7±1.8 | 1.2±0.2 | Probable citrate synthase II CitA |
| 1297 | *rho* | 1.4±0.7 | 0.5±0.2 | Probable transcription termination factor Rho homolog |
| 1803c | *PE_PGRS32* | 1.1±0.4 | **3.2±4.0** | PE-PGRS family protein |
| 3596c | *clpC1* | 1.6±0.3 | **2.3±0.8** | Probable ATP-dependent protease ATP-binding subunit ClpC1 |
| 3731 | *ligC* | **2.1±0.5** | **3.6±0.5** | Possible ATP-dependent DNA ligase LigC |
| 3757c | *proW* | 0.9±0.3 | 1.1±1.0 | Possible osmoprotectanttransport integral membrane protein ABC transporter ProW |
| 3820c | *papA2* | 1.6±0.3 | **2.4±0.4** | Possible conserved polyketide synthase associated protein PapA2 |
| 0711 | *atsA* | 0.3±0.1 | 2.0±1.2 | Possible arylsulfatase |
| 1043c |  | 1.8±0.6 | **3.4±1.4** | Conserved hypothetical protein |
| 0759c* |  | **3.3±0.2** | **2.4±1.2** | Conserved hypothetical protein |
| 2466c* |  | **482.0±158.7** | **118.9±40.2** | Conserved protein |

Values are the ratio of expression of heat or plumbagin stressed/unstressed cells in wild type H37Rv divided by expression of heat or plumbagin stressed/unstressed cells in the ΔsigH strain. Note that Rv3731 expression ratio following heat stress (52^o^C for 15 minutes) in the original experiments was <2.

* Indicates two positive control genes that showed increased expression in wild type compared to the Δ*sigH* strain in the initial heat stress experiments.

Bold indicates expression ratios (wild type:Δ*sigH*) >2 .

All genes were analyzed in technical duplicates; genes with an expression ratio >2 were analyzed in two biological replicates.

**B.** Sequences in ChIP-Seq peak that match the SigH recognition consensus for Rv0435c and Rv3596c, which showed increased SigH-dependent expression in response to plumbagin stress but not heat stress.

| **Rv No.** | **Gene** | **SigH putative consensus sequences**  **GGAAYN-16-18-GTT** | **Distance of -35 sequence from translational start site** |
| --- | --- | --- | --- |
| 0435c |  | **GGAAT**GCCCGCGCCGGCGGGCGCG**GTT** | -442 GTG |
| 3596c | *clpC1* | **GGAAC**CGACCAGGCACCGCAAG**GTT** | -116 ATG |
| 0759c* |  | **GGAAT**GAAACCTGGGGGGGGGCC**GTT** | -116 ATG |
| 2466c* |  | **GGAAC**AGGTGCGGGGCGGCCGTG**GTT** | -76 ATG |

Y=C or T; N= A,G,C or T

* Indicates two positive control genes that showed increased expression in wild type compared to the Δ*sigH* strain in the initial heat stress experiments.
